# A spiking and adapting tactile sensor for neuromorphic applications

**DOI:** 10.1038/s41598-020-74219-1

**Published:** 2020-10-14

**Authors:** Tom Birkoben, Henning Winterfeld, Simon Fichtner, Adrian Petraru, Hermann Kohlstedt

**Affiliations:** 1 Nanoelektronik, Technische Fakultät, Christian-Albrechts-Universität zu Kiel, 24143 Kiel, Germany; 2 Materials and Processes for Nanosystem Technologies, Technische Fakultät, Christian-Albrechts-Universität zu Kiel, 24143 Kiel, Germany

**Keywords:** Electrical and electronic engineering, Semiconductors, Electronic devices

## Abstract

The ongoing research on and development of increasingly intelligent artificial systems propels the need for bio inspired pressure sensitive spiking circuits. Here we present an adapting and spiking tactile sensor, based on a neuronal model and a piezoelectric field-effect transistor (PiezoFET). The piezoelectric sensor device consists of a metal-oxide semiconductor field-effect transistor comprising a piezoelectric aluminium-scandium-nitride (Al_x_Sc_1−x_N) layer inside of the gate stack. The so augmented device is sensitive to mechanical stress. In combination with an analogue circuit, this sensor unit is capable of encoding the mechanical quantity into a series of spikes with an ongoing adaptation of the output frequency. This allows for a broad application in the context of robotic and neuromorphic systems, since it enables said systems to receive information from the surrounding environment and provide encoded spike trains for neuromorphic hardware. We present numerical and experimental results on this spiking and adapting tactile sensor.

## Introduction

Neural systems and their surroundings are in an ongoing interaction. Humans, mammals and even simple forms of living species like invertebrates are well adapted to permanently changing environments which ensures their survival^1^. Herein, sensation defines the ability to convey information by a chain of transducer stages towards the brain^2,3^. It is the somatic sensory system which plays a major role in the transmission of mechanical stimuli from the environment to the brain^2,4^. The necessary signal conversion is done by mechanoreceptors underneath the skin which are sensitive to physical distortion and therefore necessary for the perception of size, shape and consistence of objects. Some important classes of mechanoreceptors responsible for a neuronal answer to mechanical stimulation are Meissner’s corpuscles, Merkel cells and Pacinian corpuscles, which can be found in glabrous skin^5–7^. Regarding mechanical perception Edgar Douglas Adrian launched a series of seminal papers formulating the firing rate hypothesis based on a number of intriguing experiments as early as 1926. Thereby, he also established the term adaptation in the framework of biological signal processing^8–10^. Today, the adaptation to a stimulus is considered as a classical response function, which is not just restricted to the encoding of external signals into internal spiking representations. Besides the adaptation of sensory neurons to a constant stimulus, spike-frequency adaptation is also present in neurons even far from sensory systems and can rise not just through cellular^11,12^, but also network induced mechanisms^13,14^. These biological findings served as a guideline to develop a tactile sensor based on piezoelectric Al_x_Sc_1−x_N (AlScN) within the fields of neuromorphic engineering and robotics.

During the last years, a tremendous effort was made to develop different concepts regarding touch and tactile sensing. Besides traditional piezoelectric materials as for example PbZr_x_Ti_1−x_O_3_ (PZT)^15^ or BaTiO_3_^16^ these concepts range from mechanical fluid based^17^ sensors to electrical approaches based on graphene^18–21^, polymers^22–26^ , and field-effect transistors coupled capacitively with nanowires^27,28^. Moreover, the possible application of pressure sensors for smart prosthetics^29^ and their capability of connecting them directly to nerve cells were explored^30^. For a deep and comprehensive overview about recent developments and further possible applications of tactile sensors and eSkin, we refer to the following review articles by Dahiya et al*.*^31^ and Soni and Dahiya^32^. In this context we would like to highlight the piezoelectric oxide semiconductor field effect transistor (POSFET) as a tactile sensor because this device has a few features in common with the here presented PiezoFET^23,33–35^. The POSFET exploits the organic ferroelectric copolymer PVDF-TrFE in the gate stack. Because ferroelectric materials are piezoelectric, the POSFET is a stress sensitive device, too^36,37^. Via the piezoelectric effect, stress induced interfacial charges modulate the channel current of the transistor. This enables the tactile sensor function of the device. Although this fundamental working principle is the same for the POSFET and PiezoFET, we would like to emphasise that the here presented PiezoFET based on AlScN exhibits a few distinguishing features. In contrast to the organic copolymer PVDF-TrFE, AlScN is compatible with Si-CMOS technology^38–40^. This allows the development of highly compact and integrated tactile sensor units consisting of PiezoFETs and subsequent neuromorphic circuits. Moreover, the ferroelectric copolymer PVDF-TrFE typically needs an initial wake-up procedure which consists of applying an electric field of about 80–100 V/µm. Such a procedure could be unfavourable for densely packed integrated sensor arrays. The electric field is necessary for the polymer device to function fully^23,34,41^. Besides the development of single sensor devices for the conversion of stress into an electrical quantity, also a tremendous progress regarding analogue circuit design was made over the last decades^42^. This ranges from the development of biologically inspired circuits like the adaptive exponential I&F neuron^43,44^ and their integration in spiking deep neural networks^45^ to mixed signal circuits which exploit events for processing visual information^46–49^. For a review on other neuromorphic systems and circuits, as well as mimicking other senses, the reader can refer to Bartolozzi et al*.*^50^.

In the here presented concept, the piezoelectric thin film inside the gate structure of the PiezoFET responds to a mechanical deformation with a polarisation. For the encoding of the signal into spikes, a Leaky Integrate and Fire (LIF) approach is used. Here, the focus was on the development of a circuitry comprising just few components. Therefore, the LIF neuron model we use does not model the conductance of ion channels explicitly. In order to generate spikes, a negative differential resistance is used which is based on the positive feedback coupling of two transistors. This is a difference to other concepts like the spike based readout of the POSFET^35^. In the following, we present the PiezoFET in more detail. Subsequently, the entire unit including the spike generation circuit is described. This encompasses a short introduction of the used neuron model as well as numerical results on the proposed spiking and adapting circuit. Finally, experimental findings performed with the touch sensor to demonstrate the spike coding of the applied forces as well as the exponential adaptation of the system are presented.

## PiezoFET

The concept of the force sensitive PiezoFET is based on a conventional field-effect transistor platform. The signal conversion from a mechanical quantity to an electrical one is achieved by a piezoelectric material. Therefore, a c-axis oriented AlScN layer containing 27% Sc is deposited in between the control gate and the channel of the MOSFET. AlScN is chosen due to its higher piezoelectric coefficient compared to pure AlN while remaining CMOS compatible paired with lower leakage compared to most perovskite piezoelectrics^38,39,51^. Mechanical stress applied to the AlScN layer induces surface charges which in turn modulate the transistor channel current. Thus, this transistor combines the stress sensing capabilities of a piezoelectric material with the amplification of a field-effect transistor. Furthermore, because the bottom AlScN interface is only a few tenth’s nm apart from the transistor channel this configuration may result in a large signal-to-noise ratio.

**Figure 1:**
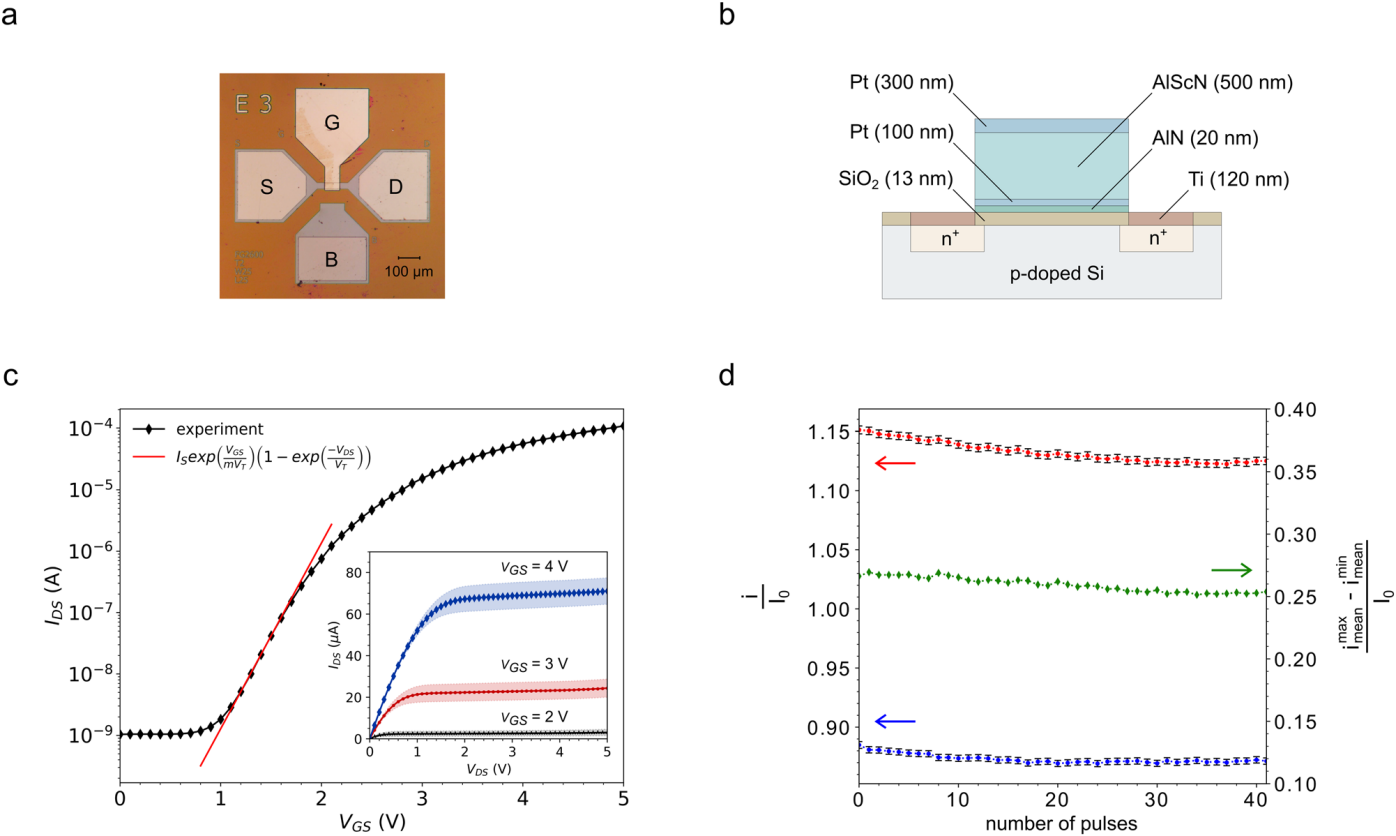
Structure and characteristics of the PiezoFET. (**a**) Microscopic image of a PiezoFET (top view). The G, S, D, and B refer to gate, source, drain, and bulk, respectively. (**b**) Schematic cross-section view of the PiezoFET. AlN and Pt function as an adhesion promoter and a seed layer for the piezoelectric thin film of AlScN. (**c**) Typical transfer curve of a PiezoFET. The device is operated in the subthreshold regime, which can be modelled by an exponential function (red line). The estimates threshold voltage is 2.75 V. The output characteristic (inset) shows the mean values for a small set of devices (N = 3) with the standard error as the shaded area. (**d**) Mean values of the current responses of a PiezoFET under periodic rectangular stimuli.

In Fig. 1a, b a microscopic image of a PiezoFET and its cross-section view are shown. The layer sequence of the gate stack from top to bottom is: Pt (300 nm), AlScN (500 nm), Pt (100 nm), AlN (20 nm) and SiO_2_ (13 nm). The AlN functions as an adhesion layer on which the Pt as a structural seed layer for the c-oriented growth of the AlScN is deposited. We assume that the influence of the 20 nm AlN layer can be neglected because it was sputter deposited directly on the amorphous gate dielectric without a seed layer in between. Therefore, it is either amorphous or has less pronounced piezoelectric parameters compared to the much thicker AlScN film. The n-MOSFET is built on a (100) oriented p-type Si and operates in the subthreshold regime leading to electrons as the majority charge carriers. The orientation of the channel regarding the crystal is in the [110] direction minimizing possible piezoresistive influences by changes in carrier mobility resulting in a changed drain current^52–55^. In Fig. 1c the transfer characteristic of the PiezoFET is shown in a semi-logarithmic plot. For gate-source voltages ranging from 0 to 2.75 V the drain-source current increases exponentially. This is the so-called subthreshold regime. The subthreshold swing of the investigated device was 330 mV/decade. The subthreshold regime represents the most sensitive region for a PiezoFET as a tactile sensor^40^. The inset of Fig. 1c shows the mean values of the drain source current for a small set of transistors. The shaded areas refer to the standard error. The drain source current in the subthreshold regime can be approximated by:

1 $$I_{DS} = I_{S} e^{{\frac{{V_{GS} }}{{mV_{T} }}}} \left( {1 - e^{{ - \frac{{V_{DS} }}{{V_{T} }}}} } \right).$$

Besides the thermal voltage $V_{T}$, the drain source voltage $V_{DS}$ and the gate source voltage $V_{GS}$, is the drain source current $I_{DS}$ dependent on the reverse current $I_{S}$ and the slope factor $m$.

**Figure 2:**
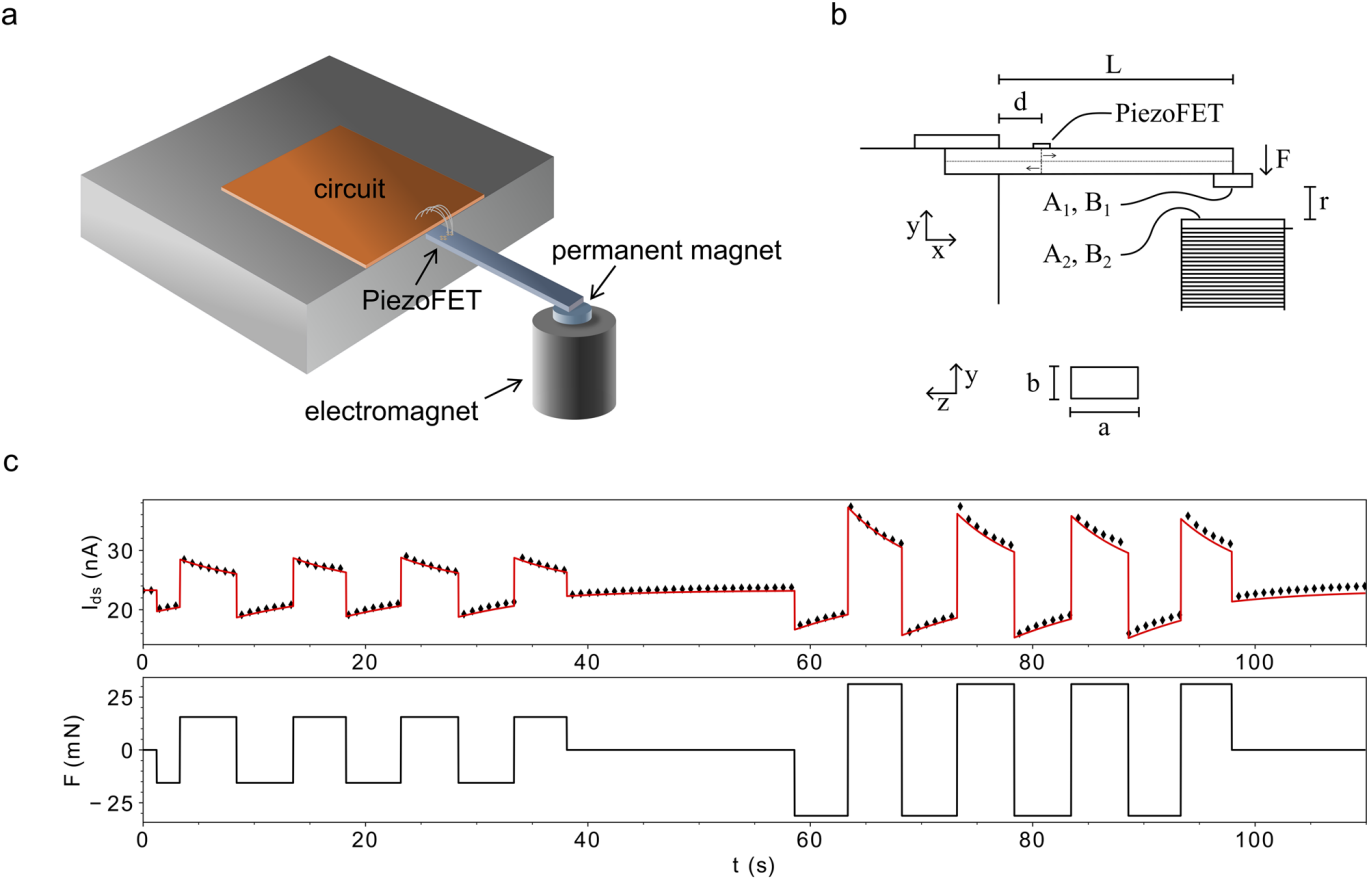
Influence of different forces on the resulting drain source current. (**a**) Experimental setup for applying a defined positive and negative force to the device in a cantilever configuration. (**b**) Definition of the used coordinate system and variables. (**c**) Experimental results during the application of different forces (black diamonds) and the numerical results obtained from Eq. (6) after fitting the IV-curve in the subthreshold regime with Eq. (1) (red line, $I_{DS}$). The time evolution of the force used for the calculation is shown at the bottom ($F$).

To verify the function of the PiezoFET, the device was integrated into a Si-cantilever configuration. The schematic 3-dimensional set-up is shown in Fig. 2a. The sketch in Fig. 2b represents the corresponding side view. The position of the sensor is crucial for the performance of the whole system, since a maximisation of stress across the transistor has a direct impact on its performance as a sensor. *F* is the force acting on the cantilever generated by a current through the electromagnet and the constant magnetic field of the permanent magnet. The force can be attractive or repulsive and arbitrary stress wave forms can be generated by appropriate bias currents. We refer to a positive force when it follows the direction indicated by the arrow in Fig. 2b. The force which deflects the cantilever can be estimated with the help of the constant magnetic field of the permanent magnet $B_{1}$, the adjustable field of the electromagnet $B_{2}$, the Area of the permanent magnet $A_{1}$, the area of the electromagnet $A_{2}$, and the permeability $\mu_{0}$. The force scales reciprocally with the quadratic distance $r$ between the permanent magnet and the electromagnet (see Fig. 2b) and reads to:

2 $$F = \frac{{B_{1} B_{2} A_{1} A_{2} }}{{4\pi r^{2} \mu_{0} }} .$$

As the force is applied to the tip of the cantilever the material bends and stress is generated.

3 $$\sigma_{11} \left( {x,y} \right) = \frac{M\left( x \right) \cdot y}{I}.$$

The amount of stress $\sigma_{11} \left( {x,y} \right)$ in the direction of the x-axis depends on the distance from clamping as well as the distance from the middle of the cantilever and is a function of the bending moment $M\left( x \right)$ and the second moment of inertia $I$^56^. For a rectangular beam the second moment of inertia can be calculated as:

4 $$I = \frac{{ab^{3} }}{12}.$$

Here, the variables $a$ and $b$ are the width and height of the cantilever, respectively. The bending moment can be found by:

5 $$M\left( x \right) = F\left( {L - d} \right).$$

The term $\left( {L - d} \right)$ defines the position of the used PiezoFET from the clamping (see Fig. 2b). With Eqs. (3)–(5) the generated voltage can be calculated using the piezoelectric coefficient $g_{31} { }$ and thickness of the piezoelectric crystal $h_{AlScN}$^57^ as follows:

6 $$V_{31} = \sigma_{11} \cdot g_{31} \cdot h_{AlScN} .$$

The piezoelectric coefficient $g_{31}$ connects the stress in x-direction with the voltage generated in the y-direction (see Fig. 2b). However, even if a constant stress is applied this voltage decays over time due to a finite leakage current, which compensates the polarisation. Therefore, the overall voltage of the piezoelectric layer $V_{P} { }$ is modelled as the time evolution of the generated voltage $V_{31}$ subtracted by its leakage. The proposed differential equation reads to:

7 $$\dot{V}_{P} = \frac{{6g_{31} h_{AlScN} \left( {L - {\text{d}}} \right)}}{{ab^{2} }}\dot{F} - \frac{{V_{P} }}{{\tau_{AlScN} }}.$$

The change in the polarisation voltage is a function of the time derivative of the force $F$ (this is $\dot{F}$) and the leakage current which compensates the generated surface charges. The characteristic time constant $\tau_{AlScN}$ regulates the current response of the system. Appling a force to the device will increase or decrease the channel current.

8 $$I_{DS} = I_{S} e^{{\frac{{V_{GS} + V_{P} }}{{mV_{T} }}}} \left( {1 - e^{{ - \frac{{V_{DS} }}{{V_{T} }}}} } \right).$$

As the piezoelectric gate layer is subjected to mechanical stress, the generated voltage is superimposed on $V_{GS}$ as the additional voltage $V_{P}$. A schematic of the experimental set up, as well as the resulting alternation of $I_{DS}$ in response to an external force are depicted in Fig. 2c. Because the applied forces and the resulting mechanical deflections are small, the distance between the tip of the cantilever and the electromagnet is considered as constant. The experimental results are compared to the results from Eqs. (12) and (13) in Fig. 2c. The black diamonds refer to experimental results from time dependent driving forces. The red line resembles the calculated force/current response of the device following well the aforementioned equations. It is well visible that an increase in the applied force also increases the drain source current, whereas the application of an opposite force decreases the current response.

## Neuron model

The observed relation between the channel current I_DS_ of the PiezoFET and the applied force, as well as its decay after applying a constant force, offers the opportunity to convert mechanical stress into neuronal-like spike trains including spike-frequency adaption. In this way, basal biological functionality can be mimicked by a compact circuitry. The circuit is based on the LIF neuron model. This model resembles basic neuronal functions, by integration of incoming information and output of spike encoded information through a firing and reset process^58,59^. The potential $x$ changes gradually with a charging current and decreases by itself. Both terms are scaled by an intrinsic time constant $\tau$ following:

9 $$\tau \dot{x} = - x + \gamma i.$$

As this system does not have a mechanism for spike generation, a threshold $x_{\vartheta }$ is defined. The spike timings refer to the exact point in time there the variable $x$ reaches this point.

10 $$t^{s} :{ }x\left( {t^{s} } \right) = x_{\vartheta } .$$

After the generation of a spike, the system is reset to its resting state $x_{r}$. The time needed for the reset process is defined as $t_{\varepsilon }$.

11 $$x\left( {t^{s} + t_{\varepsilon } } \right) = x_{r} { }{\text{.}}$$

As the integration process is repeated after reaching the reset point, the period for a constant input current is the time between two spiking events $t_{1}^{s}$ and $t_{2}^{s}$. This includes the time needed to reset $x$ once and is called the interspike interval $ISI$. A general assumption is that the discharge time is neglected, as it is much smaller than the time needed to reach the threshold.

12 $$ISI = t_{2}^{s} - t_{1}^{s} {.}$$

As mentioned earlier, in contrast to biology the reset does not generate a spike in this mathematical model. Nonetheless, from the definition of the spike times, a spike train can be derived as the sum over Dirac pulses shifted by corresponding spike times.

13 $$S\left( t \right) = \mathop \sum \limits_{i} \delta \left( {t - t_{i}^{s} } \right).$$

If a constant input current is assumed, a general solution of Eq. (9) can be derived.

14 $$x = \gamma i + \left( {x_{0} - \gamma i} \right)e^{{ - \frac{t}{\tau }}} {.}$$

From this the frequency of the model for a constant input current can be calculated as the difference for two successive spiking points. With $x_{0} = V_{rest}$ and $x = { }V_{\vartheta }$ the frequency is reciprocally proportional to the logarithm of the quotient of the firing threshold and the reset voltage of the system.

15 $$f = \frac{1}{{t_{\varepsilon } - \tau \ln \left( {\frac{{V_{\vartheta } - \gamma i}}{{V_{rest} - \gamma i}}} \right)}}.$$

## Neuronal inspired spiking tactile sensor

The coding of mechanical stress into a spiking answer is an essential step to supply neuromorphic systems with relevant environmental information. Thus, exploiting analogue circuit design might pave the way to highly integrated dense and energy-efficient systems. Furthermore, with the design of analogue oscillator circuits, with small distances to the sensor and capabilities for local information processing, complex multiplexer setups with analogue to digital converters may become obsolete. Thus, the presented system is largely bio-inspired and takes advantages of a highly efficient information processing (please see Table 1 for an overview of the constants and system parameters used).

**Table 1:** Values used for simulation and experiment.

Parameters	Value
$I_{S}$	$1.216 \times 10^{ - 12}$ A
$V_{GS}$	$1.4$ V
$m$	$5.646$
$V_{T}$	$0.025$ V
$g_{31}$	$0.039$ Vm/N
$h_{AlScN}$	$500 \times 10^{ - 9}$ m
$L$	$7.7 \times 10^{ - 3}$ m
$d$	$1.8 \times 10^{ - 3}$ m
$a$	$2 \times 10^{ - 3}$ m
$b$	$475 \times 10^{ - 6}$ m
$\tau_{AlScN}$	$8.69$ s
$R_{1}$	$9.58$ MΩ
$R_{2}$	$100$ MΩ
$R_{3}$	$100$ MΩ
$R_{l}$	$500$ MΩ
$R_{k}$	$10$ kΩ
$C_{1}$	$680 \times 10^{ - 12}$ F
$C_{2}$	$150 \times 10^{ - 12}$ F
$V_{S}$	$5$ V
$V_{th}$	$3.213$ V
$B_{1}$	$0.163$ T
$A_{1}$	$2.827 \times 10^{ - 5}$ m^2^
$A_{2}$	$2.25 \times 10^{ - 3}$ m^2^

**Figure 3:**
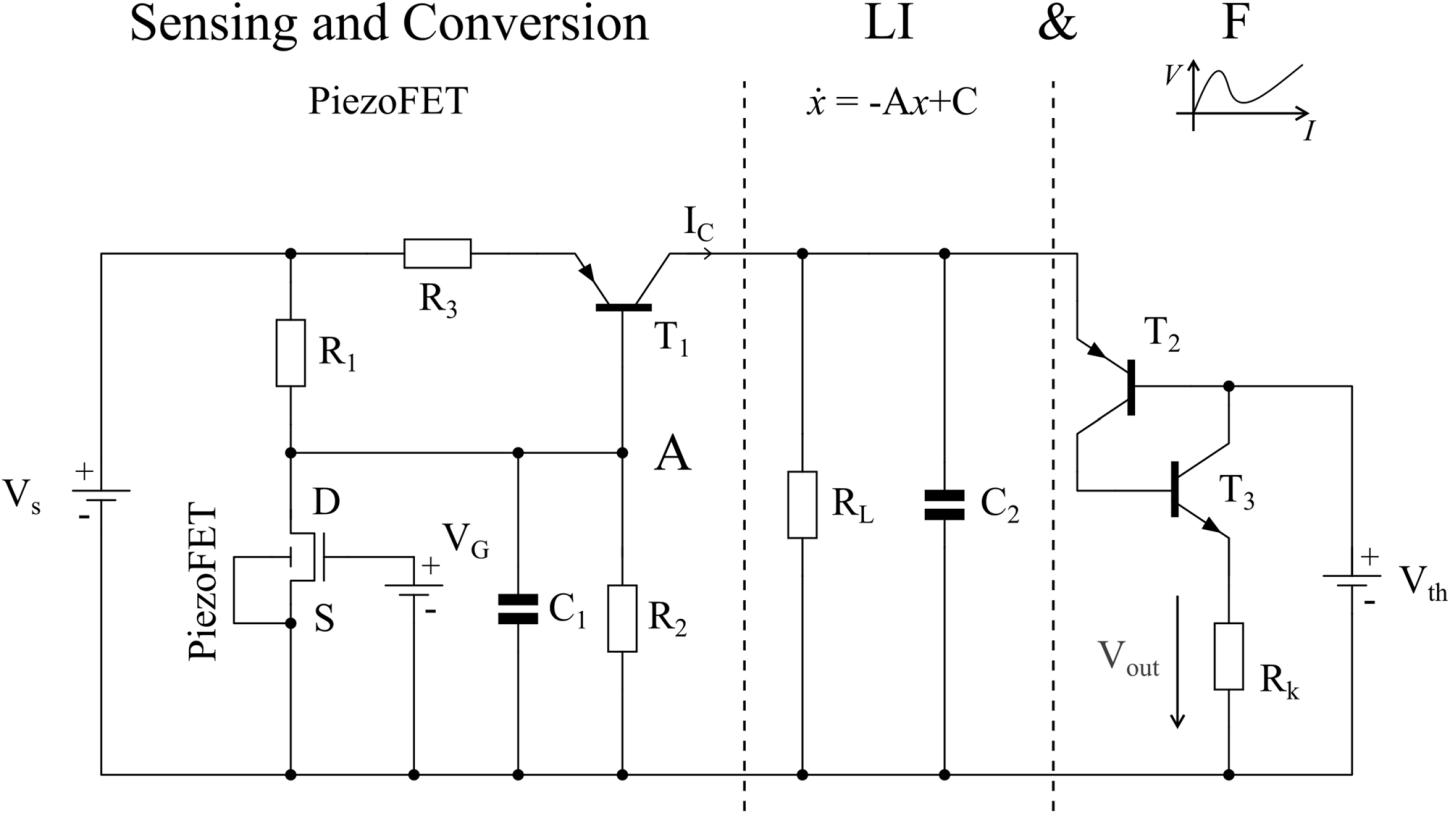
Proposed circuit as an adapting force sensor with a spike based read out. The circuit can be divided in three functional stages. The first stage is the signal sensing and conversion for the sensation of force at its conversation to a loading current for the second stage. Here, the current is integrated. When a certain voltage is reached the positive feedback loop in the third stage fires. The resulting spikes can be measured as a voltage drop over $R_{K}$ and further processed.

The circuit diagram of the adapting force sensitive analogue circuit with a spike-based readout is shown in Fig. 3. It can be divided into three functional stages. The first stage (“Sensing and Conversion”) converts the applied force into a current. It is based on the PiezoFET and is sensitive to positive and negative forces applied to the device. As stress is applied to the device, its drain source current increases significantly as can be modelled by Eq. (8). This current alters the voltage of the capacitor $C_{1}$ by influencing the voltage divider $R_{1}$ and ${\text{R}}_{2}$ directly (see Fig. 3, node “A”).

16 $$\dot{V}_{{C_{1} }} = \frac{1}{{C_{1} }}\left( {\frac{{V_{s} - V_{{C_{1} }} }}{{R_{1} }} - \frac{{V_{{C_{1} }} }}{{R_{2} }} - I_{DS} \left( {V_{P} ,V_{{C_{1} }} } \right)} \right).$$

In the definition of the proposed system, a positive force results in an increase in the current flow through the device. The increase in $I_{DS}$ will discharge the capacitor $C_{1}$ and consequently lower its voltage. The transistor ${\text{T}}_{1}$ converts this change in voltage into a current $I_{C}$.

17 $$I_{C} = I_{{T_{1} S}} \left( {e^{{\frac{{V_{EB} }}{{V_{T} }}}} - 1} \right).$$

As the transistor ${\text{T}}_{1}$ is of the pnp-type, a decrease in the voltage divider circuit will increase the voltage from emitter to base $V_{EB}$ resulting in an increased collector current $I_{C}$. This first part is followed by the second stage, the leaky integrator part (“*LI*”).

18 $$\dot{V}_{{C_{2} }} = \frac{1}{{C_{2} }}\left( {I_{C} - \frac{{V_{{C_{2} }} }}{{R_{L} }}} \right).$$

An increase in the loading current $I_{C}$ increases the voltage $V_{{C_{2} }}$, which drives the circuit to its bifurcation point. Additionally, the current influences the frequency of the resulting oscillations directly, as stated in Eq. (15).

The last stage is the firing circuit (“*F*”). This circuit is responsible for the reset to the resting potential, after reaching a threshold defined by ${\text{T}}_{2}$, ${\text{T}}_{3}$ and $V_{th}$. The transistors ${\text{T}}_{2}$ and ${\text{T}}_{3}$ build a positive feedback loop. The IV-characteristic of this pair is strongly nonlinear, with a negative differential resistance. Similar characteristics can be obtained using for example thyristors, programmable unijunction transistors or highly correlated electron materials such as VO_2_^60–62^. As the applied voltage exceeds the threshold voltage of these transistors, the current increases. This discharges the capacitor $C_{2}$, generating a pulse over $R_{K}$. If the second stage of the circuit is stimulated with a constant current, high enough so that the voltage of $C_{2}$ exceeds the threshold, the circuit will generate a series of pulses with a constant frequency. We would like to emphasize, that this is the normal form of a LIF neuron (compare Eqs. (9)–(11)).

In contrast to most thyristor approaches, the gate port is at the n-layer. At this port, the threshold voltage for the spike generation is defined. With an increase in the charging current, the frequency of the oscillator circuit changes described by Eq. (15). The discharge pulses are measurable as a voltage drop over the cathode resistance $R_{K}$. Therefore, the circuit senses and encodes the applied force into a series of spiking events. This conversion also adapts to the input force as $\tau_{AlScN}$ determines the time until the leakage current compensates the applied force.

## Simulation and experiment

**Figure 4:**
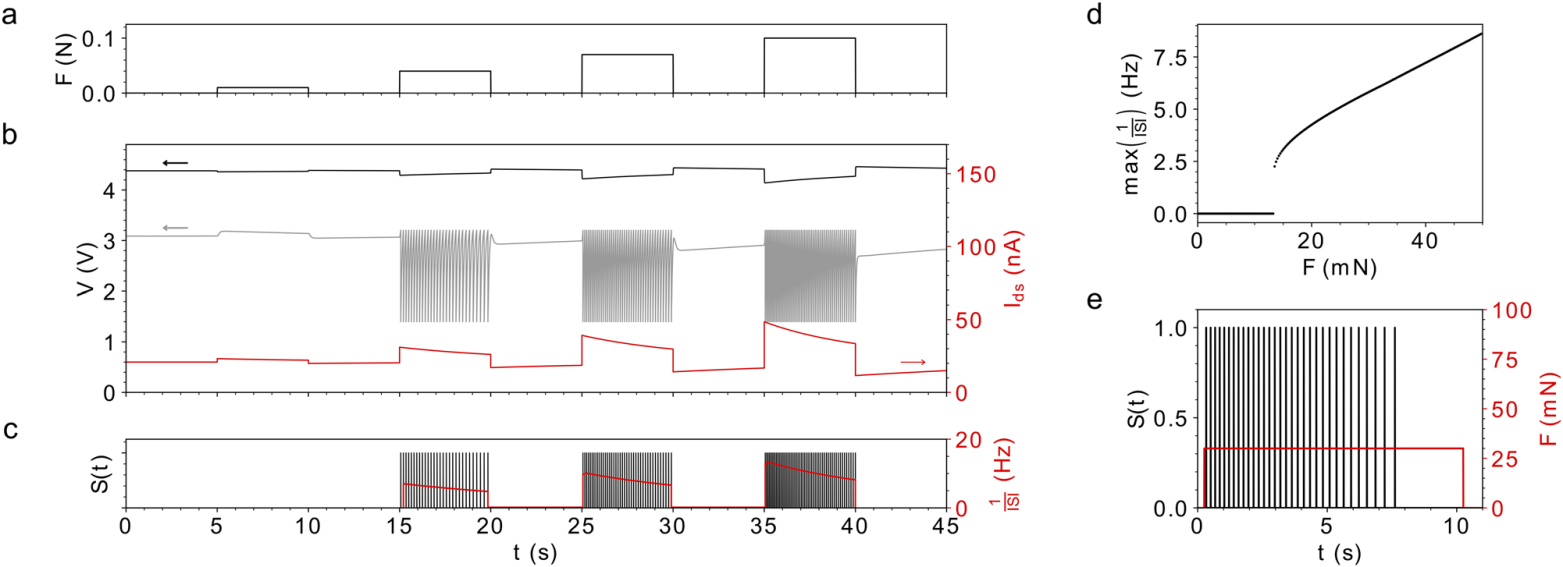
Numerical results of the proposed force sensor. (**a**) The driving force acting on the sensor, it serves as the input to the system. (**b**) The time evolution of the intrinsic voltages of the circuit and the influence a changing drain source current in the PiezoFET has on them (black: $V_{{C_{1} }}$; grey: $V_{{C_{2} }}$). The increase in the sensing current leads to ongoing oscillation of the circuit after a threshold is reached. (**c**) Resulting spike train as the system changes its stability. The resulting instantaneous firing frequency is shown in red. (**d**) Shows the maximum response frequency as a function of the applied force. (**e**) Depicts the adaptation in frequency in response to a constant driving force.

In the following, the aforementioned equations and considerations are used to simulate the overall system. The equations are solved numerically using forward integration. The results are summarized in Fig. 4. During one set of simulation, the system was stimulated with four pulses of different magnitudes. The first diagram shows the driving force as the input to the system (Fig. 4a). Below, the time evolution of the intrinsic voltages as well as the calculated current response of the PiezoFET (Fig. 4b) are depicted. In the diagram shown in Fig. 4c the resulting spike train and the corresponding instantaneous firing frequencies are shown. It is well visible, that an increase in the force increases $I_{DS}$ which results in the decrease of the voltage $V_{{{\text{C}}_{1} }}$, (Fig. 4b black trace). This lower level of the voltage, increases the charging current of the leaky integrate unit. Thus, the increase of the voltage $V_{{C_{2} }}$ of the capacitor in the second stage can be observed (Fig. 4b grey trace). The influence of the threshold voltage of the firing stage can be seen clearly, as the voltage pulses are just generated after the applied force exceeds a crucial level. Figure 4d shows the maximum frequency of the resulting spikes as a function of the driving force. During the onset of the spiking, the system changes its stability from the fixed point to a relaxation type oscillation. Thus, an increase in force also increases the frequency as the current increases as well. Because of the intrinsic compensation current of the device, the polarized layer inside the field-effect transistor will depolarize and consequently adapt to the presented stimulus. Thus, the frequency response will decrease over time. In Fig. 4e a small stimulus with a constant force is presented for 10 s. After the onset of the oscillations the frequency decreases until the oscillations vanish. This adaptation to the constant driving force is due to the depolarization of the build-up charge of the piezoelectric AlScN layer as a result of a leakage current.

**Figure 5:**
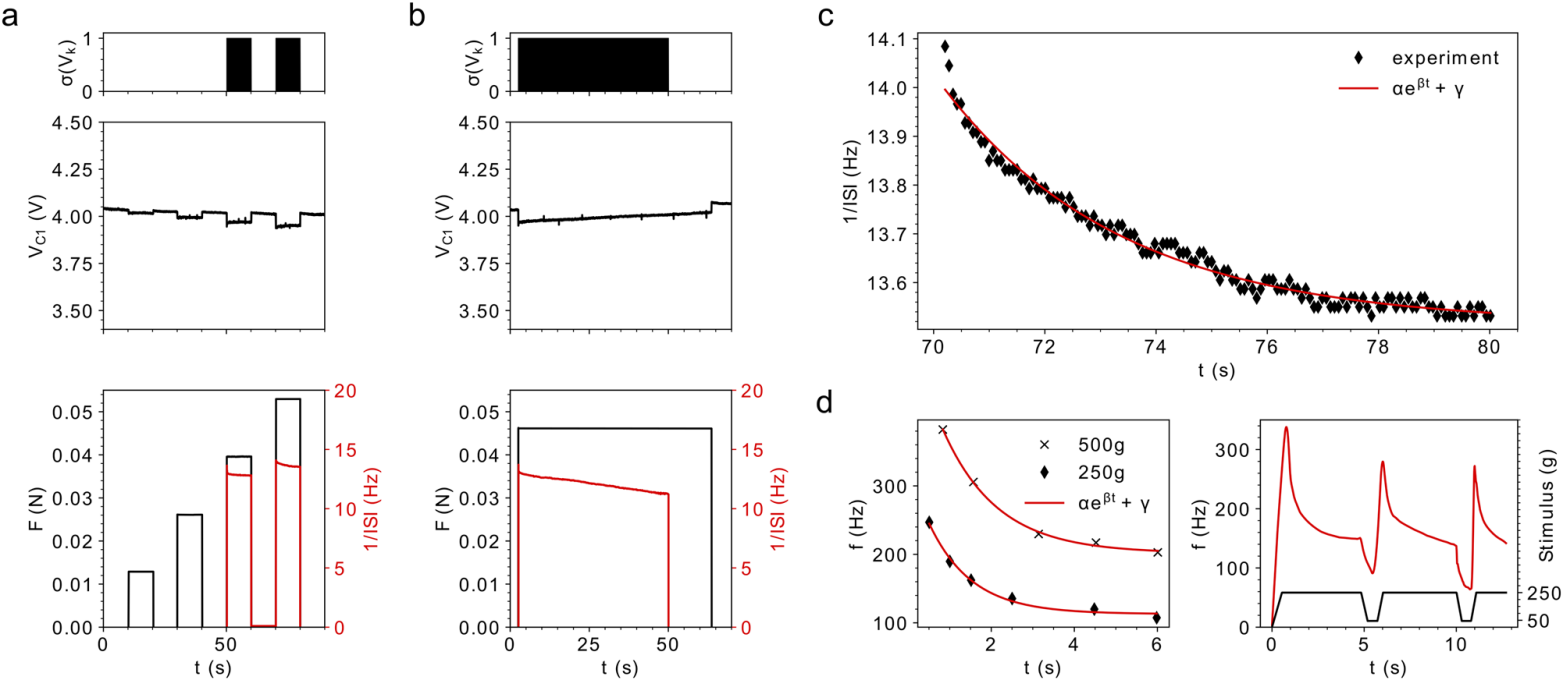
Experimental results of the spiking and adapting tactile sensor. (**a**) $\sigma \left( {V_{k} } \right)$ refers to the spike train from the application of different forces to the sensor. $V_{{C_{1} }}$ decreases as a result of the stimulating force. The transient of the applied force ($F$), as well as the instantaneous frequency ($1/ISI$) is shown. (**b**) Same time evolutions as in a), but with a different stimulation. The force was one long pulse. The adaptation is well visible. (**c**) The black diamonds refer to the instantaneous frequency of the spikes which result from a constant force. The decay in frequency is exponentially^9,64^. (**d**) Results from Adrian’s seminal experiments 1926^64^. The influence of different stimuli strengths on the spiking frequency is shown left. The dynamical system answer under an ongoing presentation of pulses is shown right (used fitting coefficients rounded to the third decimal place: $\alpha_{{500\,{\text{g}}}} = { }341.6,{~}\beta_{{500\,{\text{g}}}} = { } - 0.763,{~}\gamma_{{500\,{\text{g}}}} = { }201.679,{~}\alpha_{{250\,{\text{g}}}} = 216.413,{~}\beta_{{250\,{\text{g}}}} = - 0.978,{~}\gamma_{{250\,{\text{g}}}} = 112.859$, replot of the results from E. Adrian and Y. Zotterman^64^ ).

To verify the theoretical considerations, the system was built with discrete components on a printed circuit board. The PiezoFET was wire bonded to the circuit. Figure 5 shows the excitation of the cantilever with different forces and the resulting voltages and spike events. The spike identification was done applying a software filter.

19 $$\sigma \left( x \right) = \left\{ {\begin{array}{*{20}c} {1, \left( {x - \vartheta } \right) \ge 0} \\ {0, \left( {x - \vartheta } \right) < 0.} \\ \end{array} } \right.$$

A small threshold was used to shape the measured signal. The onset of a spiking event is marked as “1”, the absence as “0”. The results from various experiments are depicted in Fig. 5. According to the simulation, forces with different amplitudes were applied to the sensor during the first experiment. The decrease in the capacitor voltage as well as the onset of oscillation just after exceeding the threshold are visible (Fig. 5a). In addition, the adaption to the input pulses is visible as well. In the second experiment, the duration of the externally applied force was prolonged significantly. This allows the system to adapt to the input force longer. After approximately 50 s, the spike generation vanishes, although the force has still an influence on the piezoelectric field-effect transistor (see Fig. 5b).

To illustrate the adaption of the firing rate in the artificial receptor, the interspike intervals were analysed. The decay in the firing frequency was fitted by an exponential function. During the experiment an excitation force of approximately 50 mN was applied. It is visible that the exponential decay fits the experimental data. Figure 5d depicts some of the seminal work of E. Adrian on the neuronal response to mechanical stimulation. During one experiment, he studied the influence different stimuli have on the frequency response of touch and pain sensitive nerve endings.

The dependence of the frequency in relation to different intensities is shown. The dependence of the frequency response of the nerves regarding the strength of the stimulus is clearly visible. An increase in the stimulus will also increase the response. Nonetheless, the highest firing frequency is reached right after the onset of the stimulus and decays exponentially in time. This general behaviour is not just present during one excitation of the nerves but also during successive presentations of external stimuli. Although the intensity of the stimulus does not change, the maximum frequency response does decrease with each ongoing presentation (see Fig. 1d).

## Conclusion and discussion

In the first experiments carried out by Adrian et al., the exact differentiation between individual specialized cells and mechanisms was hardly possible. However, the basal biological mechanism of stimulus adaptation in a sensory system was well observable. Inspired by this seminal work, we developed a system for the encoding of mechanical stress induced through an applied force into a spiking response of a leaky integrate and fire neuron model. The depolarization of the build-up charge of the piezoelectric AlScN layer due to leakage currents, offered the opportunity to incorporate the biological important process of adaption in the sensory unit. Furthermore, the numerical and experimental findings are in good agreement. Interestingly, the presented concept and materials allow integration in Si-technology. Thus, densely packed sensor arrays for artificial spiking neural networks or oscillator-based information processing may be realized. Although, the results presented here are based on the bending of a cantilever which results in a horizontal stress on a piezoelectric material by applying a vertical force, the system could be used in biologically inspired force sensor systems as the theoretical considerations are based on the stress to which the device is subjected. Furthermore, a combination with other effects like piezoresistive modulated transistor responses might be useful and feasible as well^54,55,63^. Thus, we think that this work is a promising step towards the integration of spiking sensor units into dense interconnected systems as AlScN is a Si-CMOS compatible material^38–40,51^. Instead of using cantilevers, also planar chips with numerous PiezoFETs are feasible for spatial resolved tactile sensor units. The integration of higher numbers of adapting sensors can for example be used as an input for pattern recognition systems as the local activity changes with a variation in the applied force. The straightforward and technically simple implementation of this biologically essential adaptation process into the sensory unit, offers interesting perspectives as an interface stage for linking neuromorphic computing systems with tactile stimuli from the environment.

## Methods

The n-channel FET comprises a 13 nm thick SiO_2_ gate oxide and a piezoelectric gate stack of AlScN. The drain and source terminals, as well as the 13 nm SiO_2_ gate dielectric were fabricated in a fabline at ISIT (Fraunhofer Institute for Silicon technology, Itzehoe, Germany). The further gate stack was deposited and pattern at Kiel University. In order to archive the columnar growth of the AlScN layer, a Pt seedlayer is used. The AlN layer acts as an adhesion layer for the Pt on the SiO_2_. As a gate electrode Pt is used as well. All layers of the gate stack are deposited over the whole sample by using pulsed reactive sputtering for AlScN and DC sputtering for Pt, before the gate structure is later defined by ion beam etching. More details on the deposition of the AlScN can be found in Fichtner et al.^38^.

Following the processing of the transistor, the wafer is mechanically ground to a thickness of about 475 µm and a 2 mm × 10 mm cantilever is cut from the sample. The position of the transistor is crucial for the performance of the whole system, since a maximization of stress across the transistor directly impacts its performance as a sensor. Therefore, the transistor is located near the point at which the cantilever is fixed.

The circuit was built with freely commercially available discrete components on a printed circuit board (PCB). The circuit was designed with Autodesk Eagle. The PCB is based on an epoxy base. The base is coated with 35 µm of copper and positive photo-resist. The copper was etched using an iron(III) chloride solution. The components used to build are out of the shelf transistors and resistors. The used transistors are from the type 2N3906 and 2N3904. The voltage follower used to measure the voltages was built with a general purpose JFET amplifier of the type TL074.

The core of the magnetic coil is based on steel from the type 10025-2:2004–10. The core is 100 mm in length and 15 mm in width, forming a long bar with a square base. The coil around the steel is made of 280 turns of varnished copper with a diameter of 0.28 mm. The resulting magnetic field was measured with the teslameter 51662 from Leybold. This data is used to calculate the resulting force, during driving the coil with certain currents.

The IV-characteristic of the PiezoFET was measured with a HP 4145A Semiconductor Parameter Analyzer, without the application of an external magnetic field. The permanent magnet at the tip of the cantilever is of the typ NdFeB (N50) with a diameter of 6 mm and a thickness of 1 mm resulting in a weight of approximately 0.21 g. The surface is covered in nickel. The magnet was glued to the tip of the cantilever. During the application of force to the cantilever, all voltages were recorded with a Picoscope 3404D.

The simulation was done using Python 3.7 under the use of the libraries Numpy and Scipy for numerical calculations and Matplotlib for visualization. The piezoelectric coefficient $d_{31}$ for the material was determined experimentally. The used values for $I_{S}$, $m$ and $\tau_{AlScN}$ were received by curve fitting experimental data. The magnetic field of the permanent magnet $B_{1}$ was measured directly. The values for $B_{2}$ were determined by measuring the field of the electro magnet with different supply voltages. During the experiment the applied voltage was tracked and the corresponding magnetic field was calculated from the fitted curve.

## Data Availability

The datasets generated during and/or analysed during the current study are available from the corresponding author on reasonable request.
